# Influence of Magnetohydrodynamics and Viscosity Ratio on Modified Tetra-Hybrid Nanofluid Flow under a Threshold Non-Fourier Heat Flux Framework

**DOI:** 10.1007/s44007-026-00249-2

**Published:** 2026-09-23

**Authors:** Sachhin Sudha Mahanthesh, Mahabaleshwar Ulavathi Shettar, Laura Milenas Pérez, Laura Miller, Reinaldo Rodríguez-Ramos, Raimondo Penta

**Affiliations:** 1 Department of Mathematics and Statistics, Faculty of Natural Sciences, M S Ramaiah University of Applied Sciences, Peenya campus, Bangalore, 560 058 India; 2 Department of Studies in Mathematics, Davangere University, Shivagangothri, Davangere, 577 007 India; 3 Departamento de Ingeniería Industrial y de Sistemas, Universidad de Tarapacá, Casilla, Arica, 7D Chile; 4 Department of Mathematics and Statistics, University of Strathclyde, Livingstone Tower, 26 Richmond Street, Glasgow, G1 1XH UK; 5 Facultad de Matemática y Computacion, Universidad de La Habana, San Lázaro y L, Vedado, La Habana, 10400 Cuba; 6 PPG-MCCT, Universidade Federal Fluminense, Rio de Janeiro, 27255-125 Brasil; 7 School of Mathematics and Statistics, University of Glasgow, Mathematics and Statistics Building, University Place, Glasgow, G12 8QQ UK

**Keywords:** Magnetohydrodynamics, Tetra-hybrid nanofluid, Cattaneo–Christov heat flux, Thermal radiation, Porous media, Stretching surface

## Abstract

This study examines the combined effects of magnetohydrodynamics (MHD), viscosity ratio, and thermal radiation on the flow and heat transfer of a water-based tetra-hybrid nanofluid containing Gold, Silver, Titanium dioxide, and Aluminium oxide nanoparticles over a stretching surface. The governing momentum and energy equations are reduced to ordinary differential equations via similarity transformations, and closed-form analytical solutions are obtained using Appell hypergeometric functions. Key parameters, including the viscosity ratio, nanoparticle volume fraction, and inverse Darcy number, are analysed and compared across di-, tri-, and tetra-hybrid nanofluids. The results show that stronger magnetic fields and higher nanoparticle loadings suppress the velocity field, while changes in the thermal parameters modify the temperature distribution and thermal boundary-layer thickness. Among the formulations considered, the di-hybrid nanofluid exhibits the largest normalized wall-temperature gradient, whereas the tetra-hybrid formulation exhibits higher temperature profiles under the parameter ranges studied.

## Introduction

Porous media are made up of a solid matrix comprising various pore structures with an interconnected fluid flow. Porous media can be modelled via the Theory of Poroelasticity [1–6]. Porous media have various topographical and morphological properties based on the internal organization of the pores. Their mechanical and transport behaviour can be described using a range of continuum models, including Darcy and poroelastic formulations, each appropriate for different pore-scale regimes. These materials have many applications in contemporary technology, such as electronics cooling, catalytic reactors, packed bed heat exchangers, drugs, drug delivery systems, biological tissues, rocks, soils,and peat [7–9]. Silva et al. [10] explored the influence of a porous medium in a power law fluid that is flowing on a surface. A wide body of work has examined how permeability, microstructure, and rheology influence flow behaviour in such media, including studies on power law fluids [10], penalisation based Navier–Stokes approaches [11, 12], and turbulent transport in porous channels [13, 14].

Viscosity ratios are commonly used to characterise changes in momentum transport arising from differences in effective viscous resistance between a fluid system and a chosen reference fluid. In porous-medium flow, such effective viscosity parameters can influence the rate of momentum diffusion and the associated boundary-layer structure. In the present formulation, increasing the viscosity ratio reduces the velocity-decay parameter, leading to a slower decay of the velocity profile and hence larger velocities within the momentum boundary layer. Many researchers have studied the viscosity ratio, including Durlofsky et al. [15], who used Stokesian dynamics to study Green’s functions for flow over porous media, and Lin [16], who analysed modified Reynolds equations for squeezed film lubrication. Afterward, Harris [17] studied the fluid flow close to the stagnation points across vertical sheets with velocity slip. Further developments include stagnation point flows with slip [17], natural convection in porous vertical surfaces [18], and hybrid nanofluid transport incorporating Brownian motion and thermophoresis [19, 20].

Thermal radiation has many applications such as heat transfer by radiation in thermal imaging, which is the technique of capturing and displaying the infrared radiation emitted by objects. Its influence has been explored in contexts ranging from thermal imaging to industrial heat exchange systems, and it is often modelled using Rosseland’s diffusion approximation. Recent studies have examined radiation effects in second grade fluids [21], alumina water nanofluids under MHD forces [22], and nanoparticle aggregation phenomena [23–25].

Magnetohydrodynamics (MHD) describes the interaction between magnetic fields and electrically conducting fluids, with applications in energy systems, metallurgy, and biomedical engineering. MHD driven modifications to momentum and heat transfer have been widely analysed, including finite element simulations of thermally coupled flows [26], constrained transport MHD formulations [27], and high order numerical solvers for magnetised boundary layer problems [28–33].

In recent years, nanofluids—especially hybrid and multi-component nanofluids—have attracted increasing attention due to their enhanced thermal conductivity and tunable rheological properties. Tetra-hybrid nanofluids, which combine four distinct nanoparticle species, have been investigated for applications ranging from non Newtonian boundary layer flows [34, 35] to bio-fluid transport [36] and concentration dependent heat transfer [37–46].

Despite this growing literature, the combined influence of viscosity ratio, MHD forces, and thermal radiation on tetra-hybrid nanofluid flow over stretching surfaces remains largely unexplored. The motivation of the present work is therefore to develop an analytically tractable model that incorporates these three effects simultaneously, enabling a systematic comparison between di-, tri-, and tetra-hybrid nanofluids. A key contribution of this study is the derivation of closed form solutions expressed in terms of Appell hypergeometric functions, providing new analytical insight into the interplay between nanoparticle composition, porous media resistance, and electromagnetic forcing. The outcomes of this analysis are applicable to advanced thermal management systems, magnetically controlled cooling technologies, and high performance porous media applications. The objective of this paper is to formulate a comprehensive theoretical model that integrates the influence of magnetic field, porous medium resistance, viscosity ratio, and thermal radiation in tetra-hybrid nanofluids. Our attention to tetra-hybrid systems stems from their modified thermophysical properties and the growing interest in how multi-component nanoparticle mixtures affect momentum and thermal transport. Additionally, a comparison of di- and tri-hybrid nanofluids is carried out to see how increasing the number of different nanoparticles impacts the results.

## Mathematical Modelling and Analytical Solutions

**Fig. 1:**
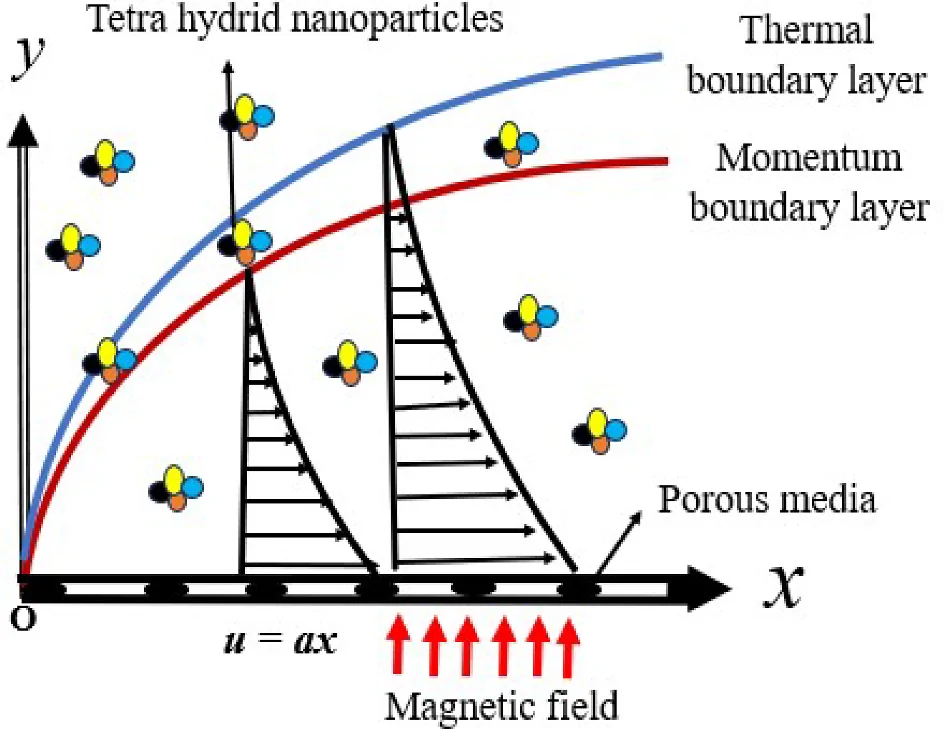
Physical configuration of the problem, showing the tetra-hybrid nanofluid flowing over a stretching surface under an applied magnetic field

The present work analyses the effect of viscosity ratio and MHD on the tetra-hybrid nanofluid flow over a stretching surface. In Fig. 1 we have a schematic representation of our structure.

The following Table 1 gives the subscripts for types of nanofluids used throughout this work.

**Table 1:** Summary of subscript notation

Symbols	Descriptions
*f*	Base fluid
*nf*	Nanofluid
*hnf*	Hybrid nanofluid
*thnf*	Ternary hybrid nanofluid
*tehnf*	Tetra-hybrid nanofluid

### Governing Equations

In this study, we consider a steady, laminar, two-dimensional, incompressible flow of a water-based tetra-hybrid nanofluid over a stretching surface embedded in a porous medium. The surface stretches with velocity u=ax, where a>0 denotes the constant stretching rate of the surface, and a uniform transverse magnetic field $B_0$ is applied. The nanofluid is treated as a homogeneous single-phase mixture with effective thermophysical properties derived from the constituent nanoparticles. Momentum transport within the porous medium is modelled using the Darcy formulation, while heat transfer incorporates both Rosseland’s diffusion approximation for thermal radiation and the Cattaneo–Christov non-Fourier heat flux model to account for thermal relaxation effects. These assumptions form the basis for the governing equations presented below using [33, 46, 47].

In vector form we have the governing equations for mass, momentum, and energy conservation for an incompressible, electrically conducting fluid

1 $$\begin{aligned} \nabla \cdot \textbf{V} = 0, \end{aligned}$$

2 $$\begin{aligned} \rho _{tehnf} \left( \textbf{V} \cdot \nabla \right) \textbf{V} = -\nabla p+\mu _{\textrm{eff}} \nabla ^{2} \textbf{V} - \frac{\mu _{tehnf}}{K}\textbf{V} + \textbf{J} \times \textbf{B}, \end{aligned}$$

3 $$\begin{aligned} {{(\rho C_{p})_{{tehnf}} \left( \textbf{V} \cdot \nabla T \right) = - \nabla \cdot \textbf{Q}}}, \end{aligned}$$

where $\textbf{Q}=\textbf{q}+\textbf{q}_r$ denotes the total non-convective heat flux, comprising $\textbf{q}$ the conductive heat flux and $\textbf{q}_r$ is the radiative heat flux. Here, $\textbf{V}=(u,v)$ is the velocity vector, *p* is the pressure, and $\rho _{tehnf}$, $\mu _{tehnf}$ and $(\rho C_p)_{tehnf}$ denote the effective density, dynamic viscosity, and volumetric heat capacity of the tetra-hybrid nanofluid, respectively. The parameter *K* is the permeability of the porous medium, while $C_{p}$ is the specific heat at constant pressure. The term $\textbf{J} \times \textbf{B}$ represents the Lorentz force due to the applied magnetic field. Here, $\mu _\textrm{eff}$ denotes the effective viscosity associated with momentum diffusion in the porous medium, whereas $\mu _{tehnf}$ denotes the effective dynamic viscosity of the tetra-hybrid nanofluid entering the Darcy resistance term. These two viscosity coefficients are therefore retained separately in the present formulation.

In the present stretching-surface configuration, the flow is driven solely by the motion of the surface, with no externally imposed pressure gradient. The pressure is therefore taken to be spatially uniform within the present model, such that $\nabla p=\textbf{0}$. Accordingly, the momentum equation reduces to

4 $$\begin{aligned} \rho _{tehnf} \left( \textbf{V} \cdot \nabla \right) \textbf{V} = \mu _{\textrm{eff}} \nabla ^{2} \textbf{V} - \frac{\mu _{tehnf}}{K}\textbf{V} + \textbf{J} \times \textbf{B}, \end{aligned}$$

The magnetic Reynolds number is defined as

5 $$\begin{aligned} Re_m = \mu _0 \sigma _{tehnf} U L, \end{aligned}$$

where $\mu _0$ is the magnetic permeability, $\sigma _{tehnf}$ is the effective electrical conductivity of the tetra-hybrid nanofluid, and *U* and *L* denote characteristic velocity and length scales, respectively.

In the present analysis, $Re_m \ll 1$, the magnetic Reynolds number is assumed to be sufficiently small, so that the magnetic field induced by the fluid motion is negligible compared with the imposed magnetic field [48]. Consequently, the imposed magnetic field may be treated as constant and the full induction problem need not be solved. Under this low-magnetic-Reynolds-number approximation, and in the absence of an externally imposed electric field, Ohm’s law for a moving electrically conducting fluid gives

6 $$\begin{aligned} \textbf{J}=\sigma _{tehnf}(\textbf{V}\times \textbf{B}). \end{aligned}$$

For the present two-dimensional flow, $\textbf{V}=(u,v,0)$, and the imposed transverse magnetic field is $\textbf{B}=(0,B_0,0)$. It therefore follows that the Lorentz force has only a streamwise component and acts in opposition to the fluid motion. Hence,

7 $$\begin{aligned} (\textbf{J}\times \textbf{B})_x =-\sigma _{tehnf}B_0^2u. \end{aligned}$$

Substituting the streamwise Lorentz-force component given by Eq. (7) into Eq. (4), and applying the boundary-layer approximation, the governing equations reduce to

8 $$\begin{aligned}&\frac{\partial {u}}{\partial {x}}{+}\frac{\partial {v}}{\partial {y}}{=}{0}, \end{aligned}$$

9 $$\begin{aligned}&u \frac{\partial u}{\partial x}+ v \frac{\partial u}{\partial y} =\frac{\mu _{\textrm{eff}}}{\rho _{\textrm{tehnf}}}\frac{\partial ^2 u}{\partial y^2}-\left( \frac{\sigma _{\textrm{tehnf}} B_0^2}{\rho _{\textrm{tehnf}}}+\frac{\mu _{\textrm{tehnf}}}{\rho _{\textrm{tehnf}} K} \right) u,\end{aligned}$$

10 $$\begin{aligned}&(\rho C_p)_{\textrm{tehnf}}\left( u \frac{\partial T}{\partial x} + v \frac{\partial T}{\partial y}\right) =- \nabla \cdot \textbf{Q} . \end{aligned}$$

The governing Eqs.  (8)–(10) involve the velocity components *u* and *v*, the temperature field *T*, and the effective thermophysical properties of the tetra-hybrid nanofluid, including its density $\rho _{\textrm{tehnf}}$, viscosity $\mu _{\textrm{tehnf}}$ and heat capacity $(\rho C_p)_{\textrm{tehnf}}$. The applied magnetic field is denoted by $B_0$, *K* represents the permeability of the porous medium, while the Darcy term models flow resistance. Together, these parameters describe the coupled effects of magnetic forces, porous-media drag, and thermal radiation on the nanofluid’s momentum and energy transport.

The boundary conditions are as follows [33, 46],

11 $$\begin{aligned} \begin{array}{c} u=u_w=ax,\ \ \ \ v=0,\ \ \ \ \ T=T_w,\ \ \ \mathrm {at\ \ \ \ }y\mathrm {=0}, \\ \ \ \qquad ~~~~u\rightarrow 0,\ \ \ ~~~~~~T\rightarrow T_\infty ,\mathrm {\ \ \ \ \ \ \ \ \ \ \ as\ \ \ \ }y\rightarrow \infty . \end{array} \end{aligned}$$

The boundary conditions reflect the physical configuration of the problem. At the stretching surface $\left( {y = 0} \right)$, the no-slip condition is imposed, such that the fluid velocity matches the surface stretching velocity u=ax, and there is no penetration normal to the surface $\left( {v = 0} \right)$. Thus, the present boundary condition corresponds to an impermeable stretching surface with no suction or injection through the wall. The temperature at the wall is maintained at a constant value ${T_w}$, representing a heated surface. As $y \rightarrow \infty$, the fluid is far from the influence of the surface, and therefore the velocity approaches zero, indicating a quiescent ambient fluid. Similarly, the temperature approaches the ambient value ${T_\infty }$, ensuring that thermal disturbances vanish far from the surface.

We now can define the parameters that describe the thermo physical properties of the tetra-hybrid nanofluids. These expressions were obtained from [35–37] and can be written as

12 $$\begin{aligned} \mu _{tehnf}&=\frac{\mu _f}{{\left( 1-\phi _1\right) }^{2.5}{\left( 1-\phi _2\right) }^{2.5}{\left( 1-\phi _3\right) }^{2.5}{\left( 1-\phi _4\right) }^{2.5}},\end{aligned}$$

13 $$\begin{aligned} {{\rho _{tehnf}}}&{{=\left( 1-\sum _{i=1}^{4}\phi _i\right) \rho _f+\sum _{i=1}^{4}\phi _i\rho _i,}}\end{aligned}$$

14 $$\begin{aligned} {(\rho C_p)_{tehnf}}&{{= \left( 1-\sum _{i=1}^{4}\phi _i\right) (\rho C_p)_f + \sum _{i=1}^{4}\phi _i(\rho C_p)_i.}} \end{aligned}$$

We now define the effective thermal conductivity and effective electrical conductivity of the tetra-hybrid nanofluid, constructed through a hierarchical mixing model. They determine how the four nanoparticle species (Au, Ag, $\text {TiO}_2$, $\text {Al}_{2}\text {O}_{3}$) modify heat and charge transport relative to the base fluid.

15 $$\begin{aligned}&\frac{\kappa _{tehnf}}{\kappa _{thnf}}=\frac{\kappa _4+2\kappa _{thnf}-2\phi _4\left( \kappa _{thnf}-\kappa _4\right) }{\kappa _4+2\kappa _{thnf}+\phi _4\left( \kappa _{thnf}-\kappa _4\right) },\end{aligned}$$

16 $$\begin{aligned}&\frac{\kappa _{thnf}}{\kappa _{hnf}}=\frac{\kappa _3+2\kappa _{hnf}-2\phi _3\left( \kappa _{hnf}-\kappa _3\right) }{\kappa _3+2\kappa _{hnf}+\phi _3\left( \kappa _{hnf}-\kappa _3\right) },\end{aligned}$$

17 $$\begin{aligned}&\frac{\kappa _{hnf}}{\kappa _{nf}}=\frac{\kappa _2+2\kappa _{nf}-2\phi _2\left( \kappa _{nf}-\kappa _2\right) }{\kappa _2+2\kappa _{nf}+\phi _2\left( \kappa _{nf}-\kappa _2\right) },\end{aligned}$$

18 $$\begin{aligned}&\frac{\kappa _{nf}}{\kappa _f}=\frac{\kappa _1+2\kappa _{f}-2\phi _1\left( \kappa _{f}-\kappa _1\right) }{\kappa _1+2\kappa _{f}+\phi _1\left( \kappa _{f}-\kappa _1\right) }. \end{aligned}$$

These expressions compute the stepwise enhancement of thermal conductivity as each nanoparticle type is added to the mixture. They determine how efficiently the nanofluid conducts heat, the thickness of the thermal boundary layer, the sensitivity of temperature to radiation effects and the magnitude of the conduction term in the energy equation. Physically, higher values lead to stronger heat diffusion and higher temperatures for the same thermal forcing.

We also have that $\sigma _1$, $\sigma _2$, $\sigma _3$, $\sigma _4$ are the electrical conductivities of the nanofluids, again using a hierarchical mixing rule. They directly control the strength of the Lorentz force in the momentum equation, the magnitude of magnetic damping under MHD effects and how strongly the magnetic parameter *M* suppresses velocity. A higher value increases electromagnetic coupling, producing stronger MHD resistance and reducing both axial and transverse velocities.

19 $$\begin{aligned}&\frac{\sigma _{tehnf}}{\sigma _{thnf}}=\frac{\left( 1+2\phi _4\right) \sigma _4+\left( 1-2\phi _4\right) \sigma _{thnf}}{\left( 1-\phi _4\right) \sigma _4+\left( 1+\phi _4\right) \sigma _{thnf}},\end{aligned}$$

20 $$\begin{aligned}&\frac{\sigma _{thnf}}{\sigma _{hnf}}=\frac{\left( 1+2\phi _3\right) \sigma _3+\left( 1-2\phi _3\right) \sigma _{hnf}}{\left( 1-\phi _3\right) \sigma _3+\left( 1+\phi _3\right) \sigma _{hnf}},\end{aligned}$$

21 $$\begin{aligned}&\frac{\sigma _{hnf}}{\sigma _{nf}}=\frac{\left( 1+2\phi _2\right) \sigma _2+\left( 1-2\phi _2\right) \sigma _{nf}}{\left( 1-\phi _2\right) \sigma _2+\left( 1+\phi _2\right) \sigma _{nf}},\end{aligned}$$

22 $$\begin{aligned}&\frac{\sigma _{nf}}{\sigma _f}=\frac{\left( 1+2\phi _1\right) \sigma _1+\left( 1-2\phi _1\right) \sigma _f}{\left( 1-\phi _1\right) \sigma _1+\left( 1+\phi _1\right) \sigma _f}. \end{aligned}$$

Here, $\phi _1$, $\phi _2$, $\phi _3$, and $\phi _4$ denote the prescribed volume-fraction parameters associated with Au, $\text {TiO}_2$, Ag, and $\text {Al}_{2}\text {O}_{3}$, respectively. The different effective thermophysical properties are evaluated using the property-specific mixture relations given in Eqs. (12)–(22).

The effective density and volumetric heat capacity are evaluated directly from the constituent volume fractions using Eqs. (13)–(14). Since these are linear mixture relations, they do not depend on an order of nanoparticle incorporation. In contrast, the effective thermal and electrical conductivities are evaluated using sequential hierarchical mixing relations. For these order-dependent relations, a fixed order is adopted throughout: species 1 is first incorporated into the base fluid to form the nanofluid (*nf*), followed successively by species 2, species 3, and species 4 to form the hybrid (*hnf*), ternary-hybrid (*thnf*), and tetra-hybrid (*tehnf*) nanofluids, respectively. Thus, the order $1\rightarrow 2\rightarrow 3\rightarrow 4$ is used consistently in Eqs. (15)–(22).

The resulting effective thermophysical properties are subsequently used to evaluate the dimensionless coefficients $A_1$, $A_2$, $A_3$, $A_4$, and $A_5$, which enter the reduced momentum and energy equations and are used throughout the analytical formulation and subsequent parametric calculations. The governing variables and dimensionless parameters employed in the model are summarised in Table 2.

**Table 2:** List of symbols and physical parameters used in the mathematical model

Symbol	Description
*u*, *v*	Velocity components in the *x*- and *y*-directions
*T*	Fluid temperature
$T_w, T_\infty$	Surface and ambient temperatures
$B_0$	Applied transverse magnetic field
*K*	Permeability of the porous medium
$\mu _\textrm{eff}$	Effective porous-medium viscosity associated with momentum diffusion
$\mu _{\textrm{tehnf}}$	Effective dynamic viscosity of the nanofluid
$\rho _{\textrm{tehnf}}$	Effective density of the nanofluid
$(\rho C_p)_{\textrm{tehnf}}$	Effective heat capacity
$\sigma _{\textrm{tehnf}}$	Effective electrical conductivity
Λ	Effective porous-medium viscosity ratio, $\mu _\textrm{eff}/\mu _f$
$Da^{-1}$	Inverse Darcy number
*M*	Magnetic parameter
*Nr*	Thermal radiation parameter
*Pr*	Prandtl number
γ	Dimensionless thermal relaxation parameter (Cattaneo–Christov model)
η	Similarity variable
f(η)	Dimensionless stream function
$\theta (\eta )$	Dimensionless temperature
*a*	Positive constant stretching rate of the surface, a>0
$\phi _i$	Prescribed volume-fraction parameter of the *i*th nanoparticle component

The following Table 3 contains the thermophysical properties, based on references [35–37], which are used to obtain the results illustrated in Sect. 3.

**Table 3:** Physical properties of the nanoliquids

Thermophysical properties	$\text {H}_{2}$O	Gold (Au)	$\text {TiO}_{2}$	Silver (Ag)	$\text {Al}_{2}\text {O}_{3}$
$\text {C}_\text {p}\left( \text {J}/\text {kgK}\right)$	4179	129	690	235	765
$\rho \left( \text {kg}/\text {m}^3\right)$	997.1	19300	4000	10500	3970
$\kappa \left( \text {W}/\text {mK}\right)$	0.613	310	8.953	429	40
$\sigma \left( \text {Sm}^{-1}\right)$	0.05	$4.1\times 10^{6}$	$1\times 10^{-12}$	$6.3\times {10}^{7}$	$1\times 10^{-12}$

### Analytic Solution for the Velocity

To obtain an explicit representation of the velocity field, we can apply similarity variables, which reduce the momentum equation to a third-order nonlinear ODE. This transformation allows the flow behaviour of the tetra-hybrid nanofluid to be captured through a single similarity function.

For a>0, we introduce the following similarity variables, as applied in [46]. We obtain *u* and *v* via the stream function given by $\psi (x, y)=(\sqrt{a\nu _f})xf(\eta )$, with $\nu _f=\frac{\mu _f}{\rho _f}$. We have

23 $$\begin{aligned} u=axf_\eta \left( \eta \right) ,\ \ \ \ \ \ \ v=-\sqrt{a\nu _f}f\left( \eta \right) ,\ \ \ \ \ \ \ \theta \left( \eta \right) =\frac{T-T_\infty }{T_w-T_\infty },\ \ \ \ \ \ \ \eta =y\sqrt{\frac{a}{\nu _f}}. \end{aligned}$$

By using the similarity variables (23), then equation (9) can be rewritten as

24 $$\begin{aligned} \Lambda \frac{d^3f}{d\eta ^3}+A_2\left( f\frac{d^2f}{d\eta ^2}-{\left( \frac{df}{d\eta }\right) }^2\right) -\left( A_3M+A_1Da^{-1}\right) \frac{df}{d\eta }=0, \end{aligned}$$

with the following associated B.Cs, as applied in [24, 37],

25 $$\begin{aligned} f_\eta \left( 0\right) =1,\ \ \ f\left( 0\right) =0,{\ \ \ f}_\eta \left( \infty \right) \rightarrow 0, \end{aligned}$$

where we have

26 $$\begin{aligned} \Lambda =\frac{\mu _\textrm{eff}}{\mu _f}, \qquad A_1=\frac{\mu _\textrm{tehnf}}{\mu _f}, \qquad A_2=\frac{\rho _\textrm{tehnf}}{\rho _f}, \qquad A_3=\frac{\sigma _\textrm{tehnf}}{\sigma _f}, \end{aligned}$$

and

27 $$\begin{aligned} M=\frac{\sigma _f B_0^2}{\rho _f a}, \qquad Da^{-1}=\frac{\mu _f}{\rho _f K a} =\frac{\nu _f}{K a}, \end{aligned}$$

where $\nu _f=\mu _f/\rho _f$ is the kinematic viscosity of the base fluid. Here, Λ denotes the effective porous-medium viscosity ratio, *M* is the magnetic parameter, and $Da^{-1}$ is the inverse Darcy number.

By using the boundary conditions Eq. (25), we obtain a solution for Eq. (24) as

28 $$\begin{aligned} f\left( \eta \right) =\frac{1}{\delta }\left( 1-e^{-\delta \eta }\right) , \end{aligned}$$

Where by using Eq. (28) in Eq. (24) we obtain

29 $$\begin{aligned} \delta =\sqrt{\frac{A_2+A_3M+A_1Da^{-1}}{\Lambda }}. \end{aligned}$$

### Analytic Solution for the Temperature

In this subsection, we derive an analytical expression for the temperature field under the Cattaneo–Christov non-Fourier heat flux model. Starting from the energy equation (10), modified via Rosseland’s approximation, we apply the similarity variables introduced in (23) to obtain a second-order linear ordinary differential equation with variable coefficients for the dimensionless temperature $\theta (\eta )$. We then show how this equation admits a threshold-type solution, following the approach of Jafarimoghaddam et al. [45] and Vishalakshi et al. [46], and express the result in terms of Appell hypergeometric functions.

Under the Rosseland diffusion approximation, and following linearisation about the ambient temperature $T_\infty$, the radiative heat flux may be written as

30 $$\begin{aligned} \textbf{q}_r=-\kappa _r\nabla T, \qquad \kappa _r=\frac{16\sigma ^*T_\infty ^3}{3\kappa ^*}. \end{aligned}$$

Consequently, the conductive and radiative contributions may be combined through the total non-convective heat flux $\textbf{Q}=\textbf{q}+\textbf{q}_r$.

In the present formulation, the Cattaneo–Christov relaxation model is applied to this total effective heat flux, giving

31 $$\begin{aligned} \textbf{Q} +\lambda \left( \frac{\partial \textbf{Q}}{\partial t} +\textbf{V}\cdot \nabla \textbf{Q} -\textbf{Q}\cdot \nabla \textbf{V} +(\nabla \cdot \textbf{V})\textbf{Q} \right) = -(\kappa _{tehnf}+\kappa _r)\nabla T . \end{aligned}$$

This modelling choice incorporates the Rosseland radiative contribution into the effective diffusive heat flux before application of the Cattaneo–Christov relaxation operator. Hence, the radiative contribution does not appear as a separate source term when the total heat flux is eliminated from the energy balance.

Substitution of the total-flux relation into the energy balance, followed by application of the similarity transformation, yields

32 $$\begin{aligned} \left( A_4+Nr\right) \frac{d^2\theta }{d\eta ^2}+A_5{Pr f\ }\frac{d\theta }{d\eta }-A_5{Pr\gamma \ }\left( f\frac{df}{d\eta }\frac{d\theta }{d\eta }+f^2\frac{d^2\theta }{d\eta ^2}\right) =0, \end{aligned}$$

where the dimensionless thermal parameters are defined by

33 $$\begin{aligned} Pr=\frac{\mu _f (C_p)_{f}}{k_f}, \qquad Nr=\frac{\kappa _r}{\kappa _f} =\frac{16\sigma ^*T_\infty ^3}{3k^*k_f}, \qquad \gamma =a\lambda . \end{aligned}$$

Here, *Pr* is the base-fluid Prandtl number, *Nr* is the thermal radiation parameter, and γ is the dimensionless Cattaneo–Christov thermal relaxation parameter.

The boundary conditions, stated in Eq. (11), are also transformed and the necessary ones to supplement (32) are as follows,

34 $$\begin{aligned} \theta \left( 0\right) =1,\qquad \theta \left( \infty \right) =0. \end{aligned}$$

We have that the coefficients $\textit{A}_{4}$ and $\textit{A}_{5}$ appearing in (32) can be defined as

35 $$\begin{aligned} \begin{gathered} A_4=\frac{\kappa _{tehnf}}{\kappa _f},\quad A_5=\frac{{\left( \rho C_p\right) }_{tehnf}}{{\left( \rho C_p\right) }_f}. \end{gathered} \end{aligned}$$

The coefficients $A_4$ and $A_5$ arise from scaling the effective thermal conductivity and heat capacity of the tetra-hybrid nanofluid by the corresponding base–fluid reference values. This ensures that the reduced energy equation is written in a fully non-dimensional form. The direct dependence of the reduced energy equation on the effective thermal conductivity and volumetric heat capacity is contained in the dimensionless coefficients $A_4$ and $A_5$. The nanoparticle composition also influences the temperature field indirectly through the velocity solution, since $A_1$, $A_2$, and $A_3$ enter the momentum equation.

To obtain a closed-form solution, we follow the threshold transformation introduced in [45, 46] and set

36 $$\begin{aligned} f\left( \eta \right) =\frac{1}{\delta }\left( 1-e^{-\delta \eta }\right) , \end{aligned}$$

where δ is a constant to be determined. Substituting this ansatz into (32) and using the velocity solution $f(\eta ) = \frac{1}{\delta }(1 - e^{-\delta \eta })$ from (28), we obtain a second-order ODE for $\theta (\eta )$ of the form

37 $$\begin{aligned} \theta _{\eta \eta } + \alpha (f)\,\theta _{\eta } = 0, \end{aligned}$$

with

38 $$\begin{aligned} \alpha \left( f\right) =A_5\left( \frac{Pr\gamma \delta f^2+\left( Pr-Pr\gamma \right) f}{\left( A_4+Nr\right) -A_5\gamma Prf^2}\right) . \end{aligned}$$

Equation (37) expresses the temperature equation in a reduced form involving the function α(f), which depends on the velocity solution f(η). To obtain a closed-form expression for $\theta (\eta )$, we integrate Eq. (37) directly and then apply the boundary conditions in Eq. (34). The resulting integrals can be simplified by introducing a change of variables from η to f(η), which transforms the nested integrals into a rational function of *f*. This structure admits an Appell hypergeometric representation, following the approach of Jafarimoghaddam et al. [45] and Vishalakshi et al. [46].

By directly integrating (37) and applying the boundary condition (34)_1_ we obtain

39 $$\begin{aligned} \theta \left( \eta \right) =1+\frac{d\theta \left( 0\right) }{d\eta }\int ^\eta _0{e^{-\int ^\eta _0{\alpha \left( f\right) d\eta }}}d\eta , \end{aligned}$$

and by applying the boundary condition (34)_2_ we obtain

40 $$\begin{aligned} \frac{d\theta \left( 0\right) }{d\eta }=-\frac{1}{\int ^\infty _0{e^{-\int ^\eta _0{\alpha \left( f\right) d\eta }}}d\eta }. \end{aligned}$$

To evaluate the nested integrals in (39) we use a change of variables and find that the innermost integral can be calculated as

41 $$\begin{aligned} -\int ^\eta _0\alpha (f)d\eta&=-\int ^{f(\eta )}_0{\frac{\alpha (f)}{(1-\delta f(\eta ))}}df(\eta )=\textrm{ln}\bigg (\frac{{(1-\delta f(\eta ))}^{-A}}{{(1-Sf(\eta ))}^{-B}{(1+Sf(\eta ))}^{-C}}\bigg ). \end{aligned}$$

where we have that the exponents *A*, *B*, *C* and parameter *S* are given by

42 $$\begin{aligned} . \begin{gathered} A=\left( \frac{A_5}{A_4+Nr}\right) \frac{Pr}{\frac{\gamma PrA_5}{A_4+Nr}-\delta ^2},\\ B=\frac{\delta \sqrt{\frac{{Pr A_5\ }}{A_4+Nr}}+\sqrt{\gamma }\left( \left( \frac{A_5}{A_4+Nr}\right) Pr+\delta ^2-\gamma Pr\left( \frac{A_5}{A_4+Nr}\right) \right) }{2\sqrt{\gamma }\left( \gamma \left( \frac{A_5}{A_4+Nr}\right) Pr-\delta ^2\right) },\\ C=\frac{-\delta \sqrt{\frac{{Pr A_5\ }}{A_4+Nr}}+\sqrt{\gamma }\left( \left( \frac{A_5}{A_4+Nr}\right) Pr+\delta ^2-\gamma Pr\left( \frac{A_5}{A_4+Nr}\right) \right) }{2\sqrt{\gamma }\left( \gamma \left( \frac{A_5}{A_4+Nr}\right) Pr-\delta ^2\right) }, \\ S=\sqrt{\frac{\gamma {Pr A_5\ }}{A_4+Nr}}. \end{gathered} \end{aligned}$$

Therefore, using (41) we can write the outermost integral from nested integrals (39) as

43 $$\begin{aligned} . \int ^\eta _0{e^{-\int ^\eta _0{\alpha \left( f\right) d\eta }}d\eta }=\int ^{f\left( \eta \right) }_0{\frac{{\left( 1-\delta f\left( \eta \right) \right) }^{-\left( A+1\right) }}{{\left( 1-Sf\left( \eta \right) \right) }^{-B}{\left( 1+Sf\left( \eta \right) \right) }^{-C}}}df\left( \eta \right) . \end{aligned}$$

Integrals of this type admit a closed-form representation in terms of the Appell hypergeometric function $F_1$, and so (43) can be solved as

44 $$\begin{aligned} .&\int ^{f\left( \eta \right) }_0{\frac{{\left( 1-\delta f\left( \eta \right) \right) }^{-\left( A+1\right) }}{{\left( 1-Sf\left( \eta \right) \right) }^{-B}{\left( 1+Sf\left( \eta \right) \right) }^{-C}}}df\left( \eta \right) \nonumber \\&=\frac{1}{A\delta }{\left( 1-\delta f\left( \eta \right) \right) }^{-A}{\left( 1-Sf\left( \eta \right) \right) }^B {\left( 1+Sf\left( \eta \right) \right) }^C{\left( \frac{\delta -\delta Sf\left( \eta \right) }{\delta -S}\right) }^{-B}\ {\left( \frac{\delta +\delta Sf\left( \eta \right) }{\delta +S}\right) }^{-C}\nonumber \\&\times {\left. F_1\left( -A;-B,-C;-A+1;\frac{S\left( \delta f\left( \eta \right) -1\right) }{\delta -S},\frac{-S\left( \delta f\left( \eta \right) -1\right) }{\delta +S}\right) \right| }^{f\left( \eta \right) }_0. \end{aligned}$$

We have the identity45This identity is used because the integral in Eq. (43) admits the Appell hypergeometric representation $F_1$ given in Eq. (44), whereas the final temperature solution is more conveniently expressed in terms of the Gauss hypergeometric function . Accordingly, the identity in Eq. (45) is applied to convert the Appell representation into the corresponding  form. Therefore, the solution obtained is46To guarantee convergence of the integral in Eq. (43) as $\eta \rightarrow \infty$, we examine the behaviour of the integrand near its upper limiting value. From Eq. (28),

47 $$\begin{aligned} f(\eta )\rightarrow \frac{1}{\delta } \qquad \text {as} \qquad \eta \rightarrow \infty . \end{aligned}$$

Hence,

48 $$\begin{aligned} 1-\delta f(\eta )\rightarrow 0. \end{aligned}$$

The integrand in Eq. (43) is

49 $$\begin{aligned} (1-\delta f)^{-(A+1)} (1-Sf)^{-B} (1+Sf)^{-C}. \end{aligned}$$

Provided the threshold condition derived below is satisfied, we have S<δ, so that the factors $(1-Sf)^{-B}$ and $(1+Sf)^{-C}$ remain finite as $f\rightarrow 1/\delta$. Therefore, the convergence of the integral is governed by

50 $$\begin{aligned} (1-\delta f)^{-(A+1)}. \end{aligned}$$

The corresponding endpoint integral converges if and only if

51 $$\begin{aligned} A<0. \end{aligned}$$

Using the definition of *A* in Eq. (42),

52 $$\begin{aligned} A= \frac{\displaystyle Pr\,\frac{A_5}{A_4+Nr}}{\displaystyle \gamma Pr\,\frac{A_5}{A_4+Nr}-\delta ^2}, \end{aligned}$$

and noting that Pr>0, $A_5>0$, and $A_4+Nr>0$, the condition A<0 requires

53 $$\begin{aligned} \delta ^2> \gamma Pr\,\frac{A_5}{A_4+Nr}. \end{aligned}$$

Since, from Eq. (29),

54 $$\begin{aligned} \delta ^2= \frac{A_2+A_3M+A_1Da^{-1}}{\Lambda }, \end{aligned}$$

the convergence condition may equivalently be written as

55 $$\begin{aligned} \gamma \Lambda < \frac{(A_4+Nr) \left( A_2+A_3M+A_1Da^{-1}\right) }{Pr\,A_5}. \end{aligned}$$

This condition also implies

56 $$\begin{aligned} S^2=\gamma Pr\,\frac{A_5}{A_4+Nr}<\delta ^2, \end{aligned}$$

and hence S<δ, ensuring that the remaining factors in the integrand of Eq. (43) remain finite at the upper endpoint.

Under this convergence condition, then we are now able to use (46), in (40) with (42) to obtain57and similarly using this result with (39) gives58where we have *A*, *B*, *C* and *S* are the same as defined in (42).

The closed–form expressions obtained in Sect. 2.3 provide a complete analytical description of both the velocity and temperature fields, incorporating the combined effects of viscosity ratio, magnetic forcing, thermal radiation, and the Cattaneo–Christov relaxation mechanism. We are now able to examine how the key dimensionless parameters influence the flow and heat transfer characteristics of di-, tri-, and tetra-hybrid nanofluids. In the following section, we present a detailed parametric study based on the derived formulas, highlighting the qualitative trends and physical implications of the model.

## Results and Discussion

For consistency in the comparative analysis of the di-hybrid, tri-hybrid, and tetra-hybrid nanofluids, the prescribed total nanoparticle loading is fixed at

59 $$\begin{aligned} \phi _{total}=\sum \limits _{i = 1}^4 {{\phi _i}} = 0.03. \end{aligned}$$

This prescribed total loading is distributed equally among the nanoparticle volume-fraction parameters present in each formulation. Accordingly, the individual nanoparticle volume fractions are ${\phi _1} = {\phi _2} = 0.015$ for the di-hybrid nanofluid, ${\phi _1} = {\phi _2} = {\phi _3} = 0.010$ for the tri-hybrid nanofluid, and ${\phi _1} = {\phi _2} = {\phi _3} = {\phi _4} = 0.0075$ for the tetra-hybrid nanofluid. Thus, all three nanofluid formulations are compared under the same total nanoparticle loading, ensuring a consistent assessment of the influence of nanoparticle composition on the flow and thermal characteristics. The paper explores the influence of radiation, viscosity ratio, and MHD on the tetra-hybrid nanofluid flow across a stretching surface. The effects of thermal radiation, viscosity ratio, magnetic forcing, porous-medium resistance, and nanoparticle loading are investigated using the analytical solutions derived in Sect. 2. The resulting velocity and temperature profiles are compared for di-, tri-, and tetra-hybrid nanofluid formulations.

**Fig. 2:**
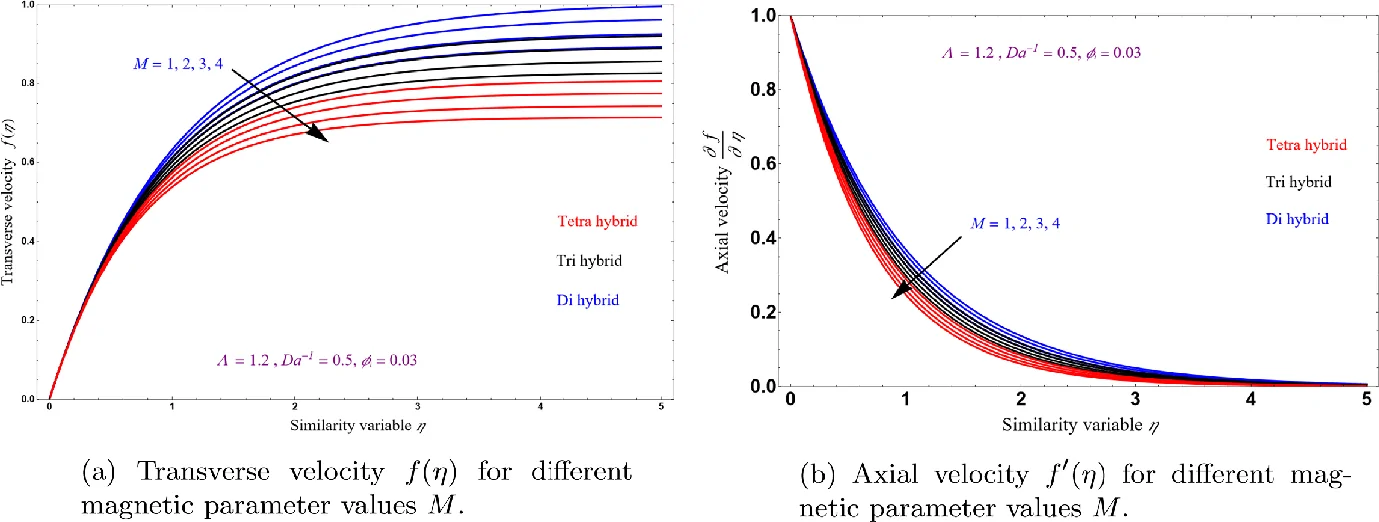
Effect of the magnetic parameter *M* on (**a**) the transverse velocity f(η) and (**b**) the axial velocity $f^{\prime }\left(\eta \right)$ for di-, tri-, and tetra-hybrid nanofluids

Figure 2 illustrates the influence of the magnetic parameter *M* on the transverse velocity f(η) (Fig. 2a) and axial velocity $f^{\prime }\left(\eta \right)$ (Fig. 2b) for di-, tri-, and tetra-hybrid nanofluids over a stretching surface. Increasing *M* strengthens the Lorentz force generated by the applied magnetic field, which opposes fluid motion and acts as a damping body force. This electromagnetic resistance reduces kinetic energy and suppresses momentum transport, producing a monotonic decrease in both velocity components across the boundary layer. A clear hierarchy emerges among the nanoparticle formulations: tetra-hybrid nanofluids exhibit the lowest velocities at all η followed by tri-hybrid and then di-hybrid fluids. The relative ordering of the three nanofluid formulations reflects differences in the effective-property coefficients $$A_1$$, $$A_2$$, and $$A_3$$, which together determine the velocity-decay parameter δ through Eq. (29). In particular, the electrical-conductivity ratio $$A_3$$ controls the contribution of magnetic forcing through the term $$A_3M$$. Near the wall, higher *M* yields steeper velocity gradients and thinner momentum boundary layers, while far from the surface all profiles converge to zero, confirming the dominance of magnetic damping away from the stretching sheet.

**Fig. 3:**
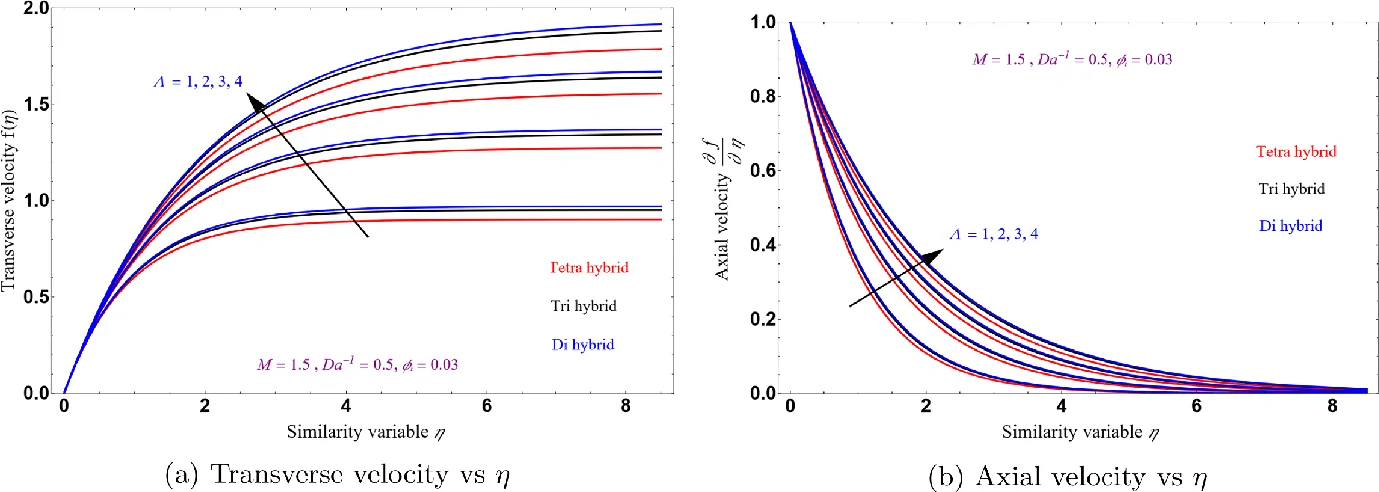
Effect of the viscosity ratio Λ on (**a**) the transverse velocity f(η) and (**b**) the axial velocity $f^{\prime }\left(\eta \right)$ for di-, tri-, and tetra-hybrid nanofluids

Figure 3 illustrates the influence of the viscosity ratio Λ on the transverse velocity f(η) and axial velocity $f^{\prime }\left(\eta \right)$ for di-, tri-, and tetra-hybrid nanofluids. From Eq. (29), the velocity-decay parameter δ varies as $$\Lambda ^{-1/2}$$ when the other governing parameters are fixed. Hence, increasing Λ decreases δ, which reduces the exponential decay rate of the axial velocity $$f'(\eta )=e^{-\delta \eta }$$. Consequently, both the transverse velocity f(η) and the axial velocity $f^{\prime }\left(\eta \right)$ increase with increasing Λ, equivalently with decreasing δ. The slower decay of $f^{\prime }\left(\eta \right)$ corresponds to a thicker momentum boundary layer. Furthermore, since $-f^{\prime \prime }\left(0\right)=\delta$, the magnitude of the wall velocity gradient decreases as Λ increases. These trends are consistent with the analytical velocity solution given by Eqs. (28)–(29).

**Fig. 4:**
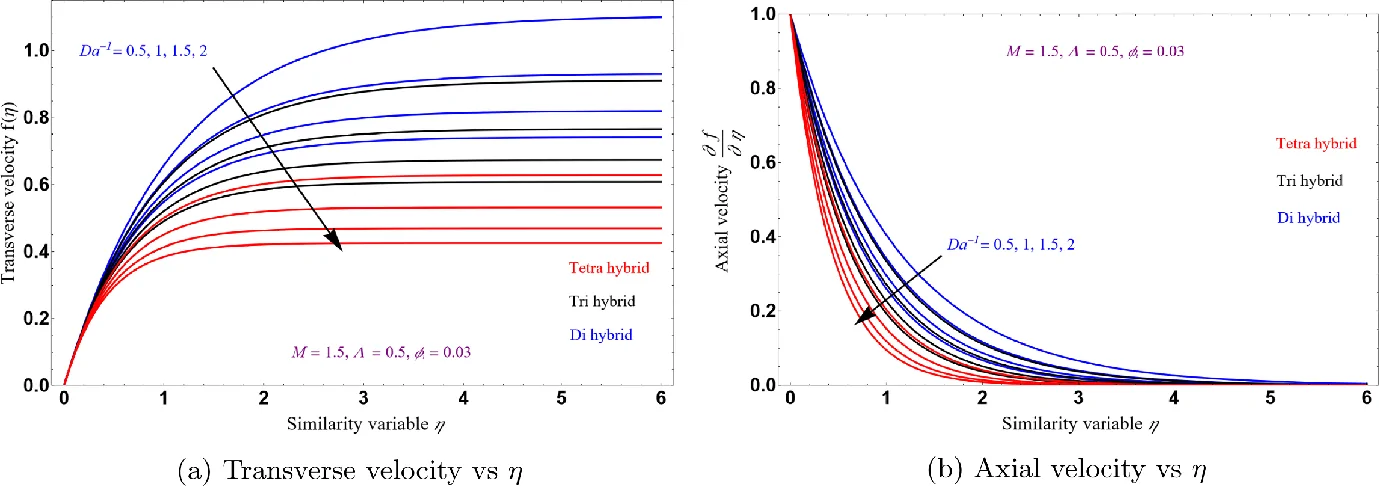
Influence of the inverse Darcy number $$Da^{-1}$$ on (**a**) the transverse velocity f(η) and (**b**) the axial velocity $f^{\prime }\left(\eta \right)$

Figure 4 presents the effect of the inverse Darcy number $Da^{-1}$ on the transverse and axial velocity profiles. Since $Da^{-1}$ quantifies the drag imposed by the porous matrix, increasing its value intensifies Darcy resistance and suppresses fluid motion. Both velocity components decrease consistently with higher $Da^{-1}$, demonstrating the dominant role of porous-media drag in attenuating momentum transport. The enhanced porous-medium resistance also increases the velocity-decay rate, resulting in a thinner momentum boundary layer and a sharper velocity gradient near the stretching surface. The relative ordering of the di-, tri-, and tetra-hybrid nanofluids remains consistent with the corresponding velocity profiles shown in Fig. 3.

**Fig. 5:**
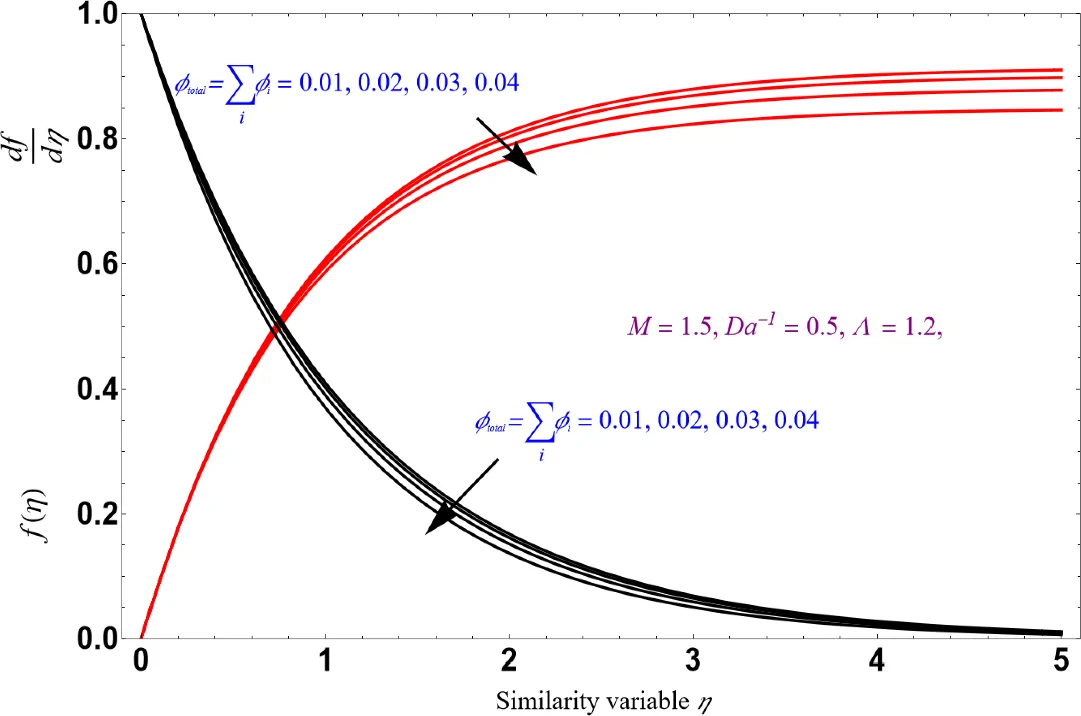
Effect of total nanoparticle volume fraction $\phi _{total}$ on the transverse and axial velocity profiles

Figure 5 shows the influence of the total nanoparticle volume fraction $\phi _{total}$ on the transverse and axial velocity distributions. Increasing $\phi _{total}$ raises the effective viscosity and density of the nanofluid, enhancing momentum diffusion and resistance to motion. As a result, both velocity components decrease steadily with increasing $\phi _{total}$, with the strongest suppression occurring near the wall. At larger values of η, all profiles converge smoothly, indicating that particle-induced resistance dominates throughout the boundary layer. These results highlight the trade-off between the changes in effective thermophysical properties associated with higher nanoparticle loading and the corresponding reduction in momentum transport.

**Fig. 6:**
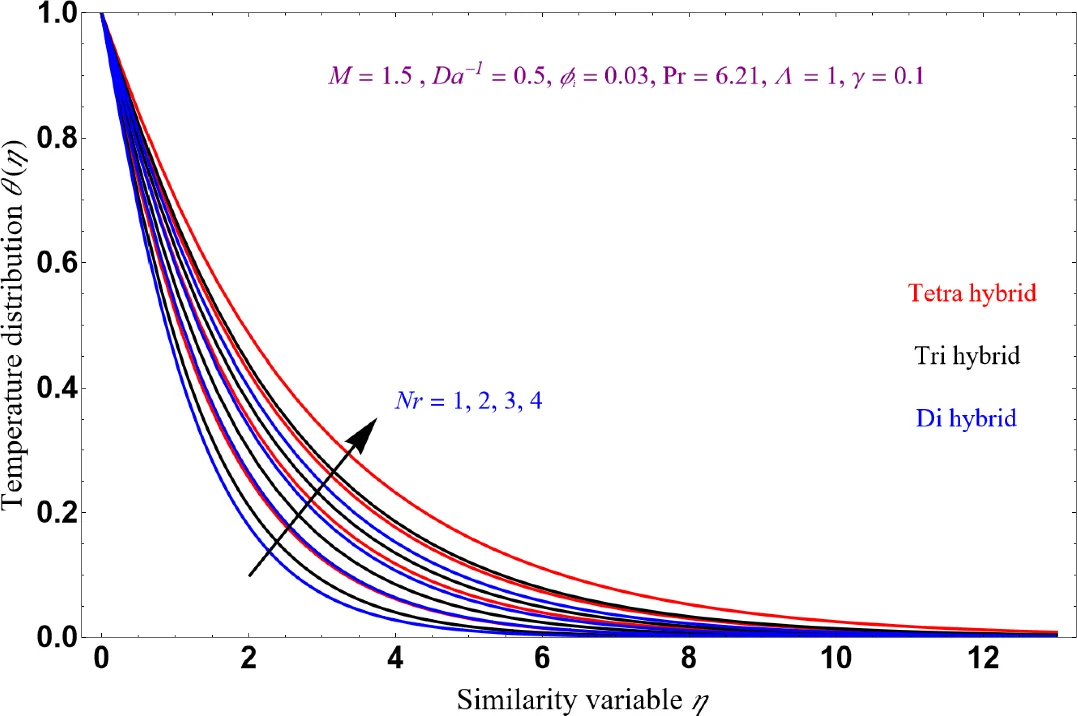
Temperature distribution $\theta (\eta )$ for varying thermal radiation parameter *Nr* in di-, tri-, and tetra-hybrid nanofluids

In Fig. 6 we have considered the effect of the thermal radiation parameter *Nr* on the temperature distribution $\theta (\eta )$ for di-, tri-, and tetra-hybrid nanofluids. Increasing *Nr* enhances radiative heat flux within the boundary layer, raising the temperature field and thickening the thermal boundary layer. Tetra-hybrid nanofluids exhibit the highest temperatures across the considered range of η, followed by tri-hybrid and di-hybrid mixtures. This ordering reflects differences in the effective thermophysical properties of the three formulations and their resulting influence on the temperature field. The monotonic increase in $\theta (\eta )$ with *Nr* demonstrates the strong coupling between radiation and thermal transport in nanoparticle-laden porous media.

**Fig. 7:**
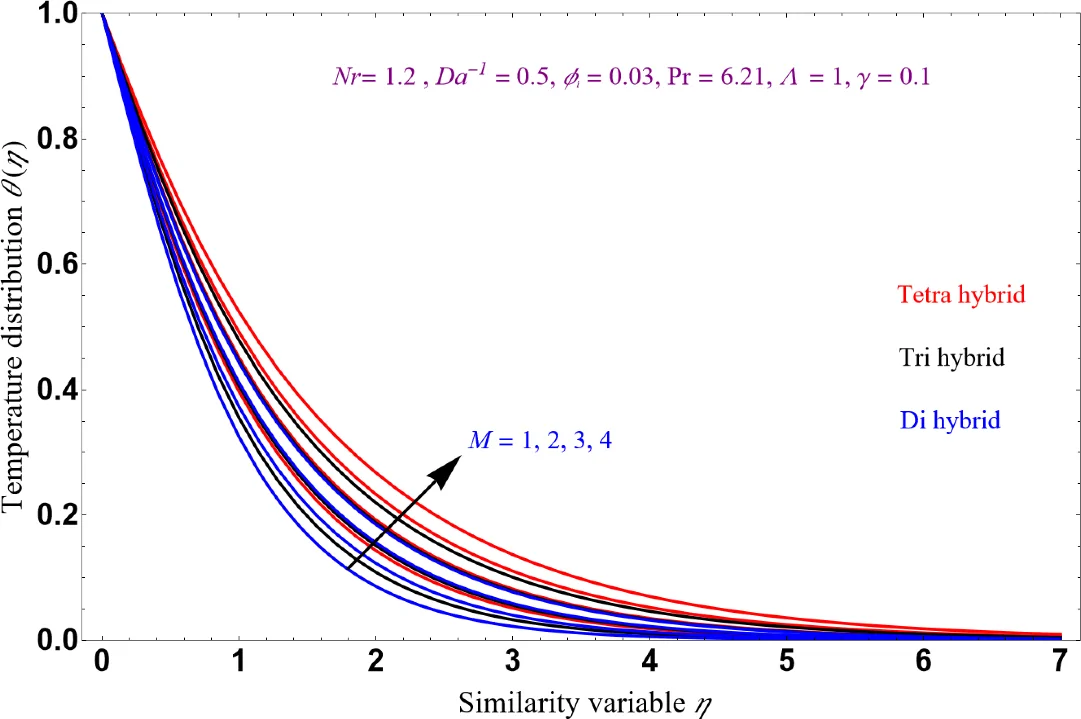
Effect of the magnetic parameter *M* on the temperature distribution $\theta (\eta )$

Figure 7 illustrates the influence of the magnetic parameter *M* on the temperature distribution $\theta (\eta )$. Increasing *M* strengthens the Lorentz force and suppresses the fluid velocity. The resulting modification of the velocity field changes the convective transport of thermal energy and consequently alters the temperature distribution. Thus, the observed variation in temperature with *M* arises indirectly through magnetic damping of the momentum field rather than from direct Joule heating, since no explicit Joule-heating source term is included in the present energy equation.

**Fig. 8:**
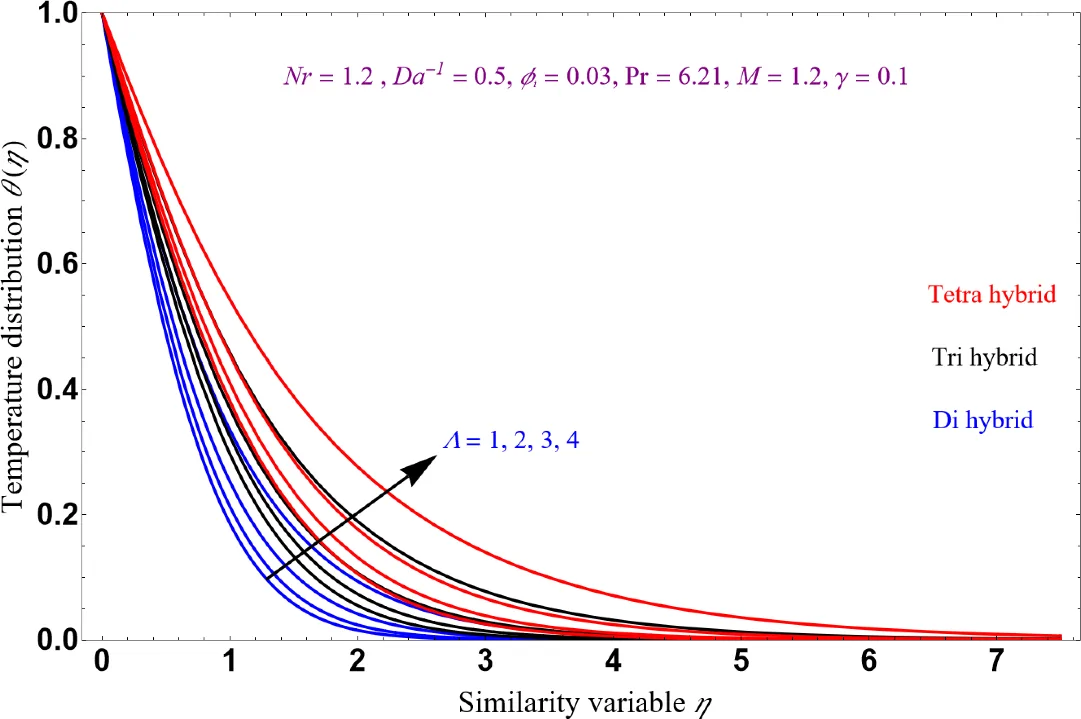
Temperature distribution $\theta (\eta )$ for varying viscosity parameter Λ

Figure 8 shows the effect of the viscosity ratio Λ on the temperature distribution $\theta (\eta )$. Increasing Λ modifies the velocity field and consequently changes the convective transport of thermal energy. Therefore, the corresponding variation in the temperature distribution arises through the coupling between the momentum and energy equations rather than from a direct viscous-dissipation heat source. Since Λ is defined as the effective viscosity ratio in the present formulation, its influence on temperature is mediated through the modified velocity field.

**Fig. 9:**
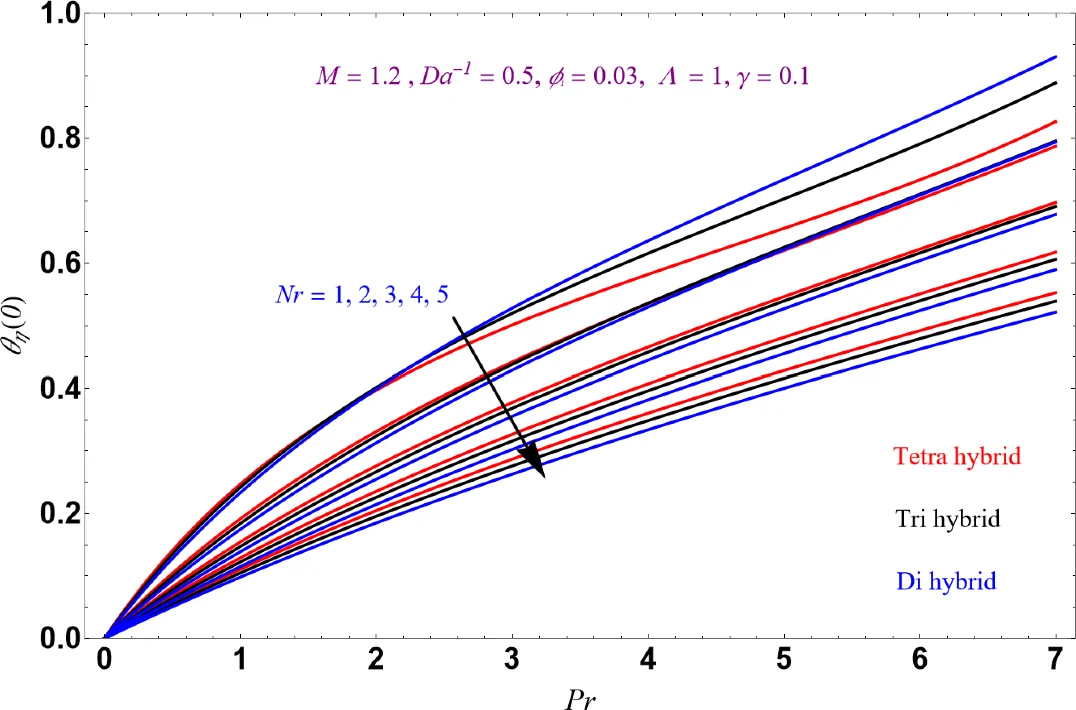
Variation of the normalized wall-temperature gradient $-\theta _{\eta }(0)$ with the Prandtl number *Pr* for different thermal radiation parameters *Nr*

Figure 9 depicts the variation of the normalized wall-temperature gradient $-\theta _{\eta }(0)$ with the Prandtl number *Pr* for different values of the thermal radiation parameter *Nr*. Increasing *Pr* reduces thermal diffusivity, producing thinner thermal boundary layers and higher normalized wall-temperature gradient. Conversely, increasing *Nr* enhances radiative heat transport, which thickens the thermal boundary layer and decreases $-\theta _{\eta }(0)$. Among the considered nanofluids, the normalized wall-temperature gradient depends on the combined effects of effective thermal properties and the temperature-field distribution. The di-hybrid nanofluid exhibits the largest magnitude of the normalized wall-temperature gradient in the present comparison, whereas the tri-hybrid and tetra-hybrid nanofluids show comparatively lower values.

The quantity $-\theta _{\eta }(0)$ is reported here solely as the normalized wall-temperature gradient and is not identified with the dimensional total wall heat flux or the Nusselt number, which would additionally depend on the effective thermal conductivity and the adopted heat-flux formulation.

**Fig. 10:**
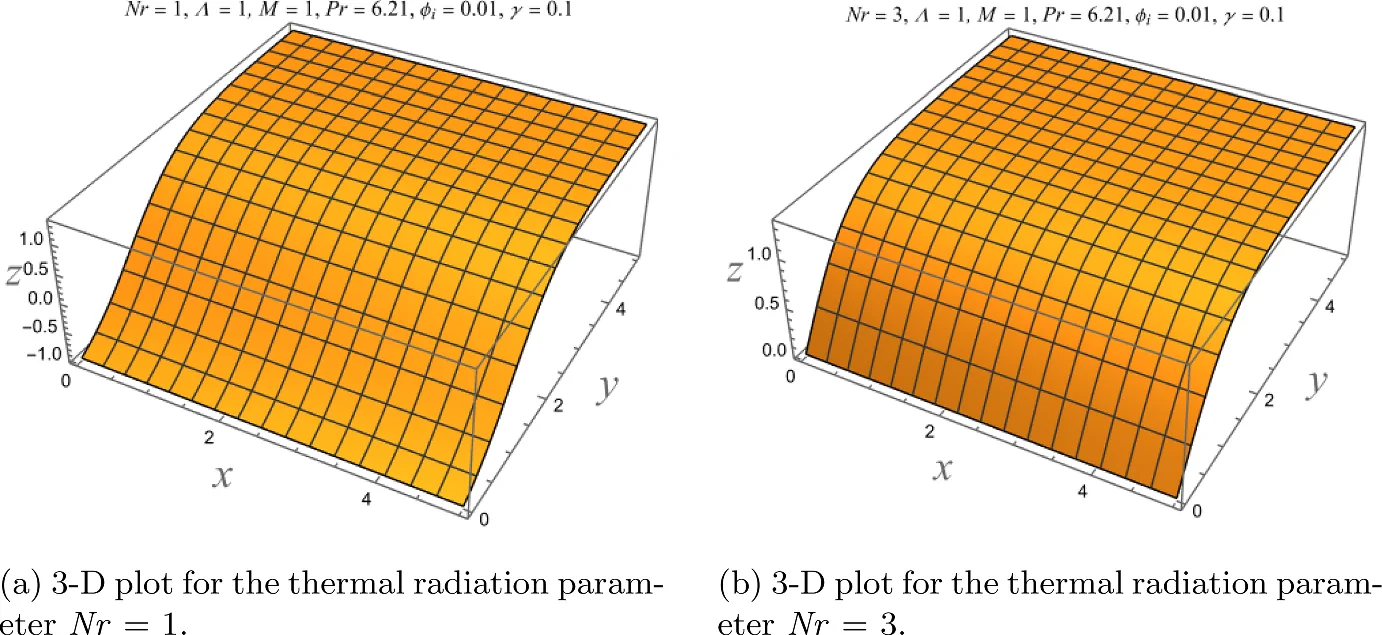
Three-dimensional temperature fields for two values of the thermal radiation parameter *Nr*

Figure 10 provides three-dimensional visualisations of the temperature field for two values of the thermal radiation parameter *Nr*. For lower *Nr*, the temperature remains concentrated near the stretching surface, with moderate gradients in both the streamwise and normal directions. When *Nr* increases, radiative heat transfer becomes more dominant, producing a visibly thicker thermal region and elevating temperatures throughout the domain. These 3-D representations illustrate how radiation modifies the global thermal structure of the flow, complementing the one-dimensional boundary-layer profiles presented earlier.

**Fig. 11:**
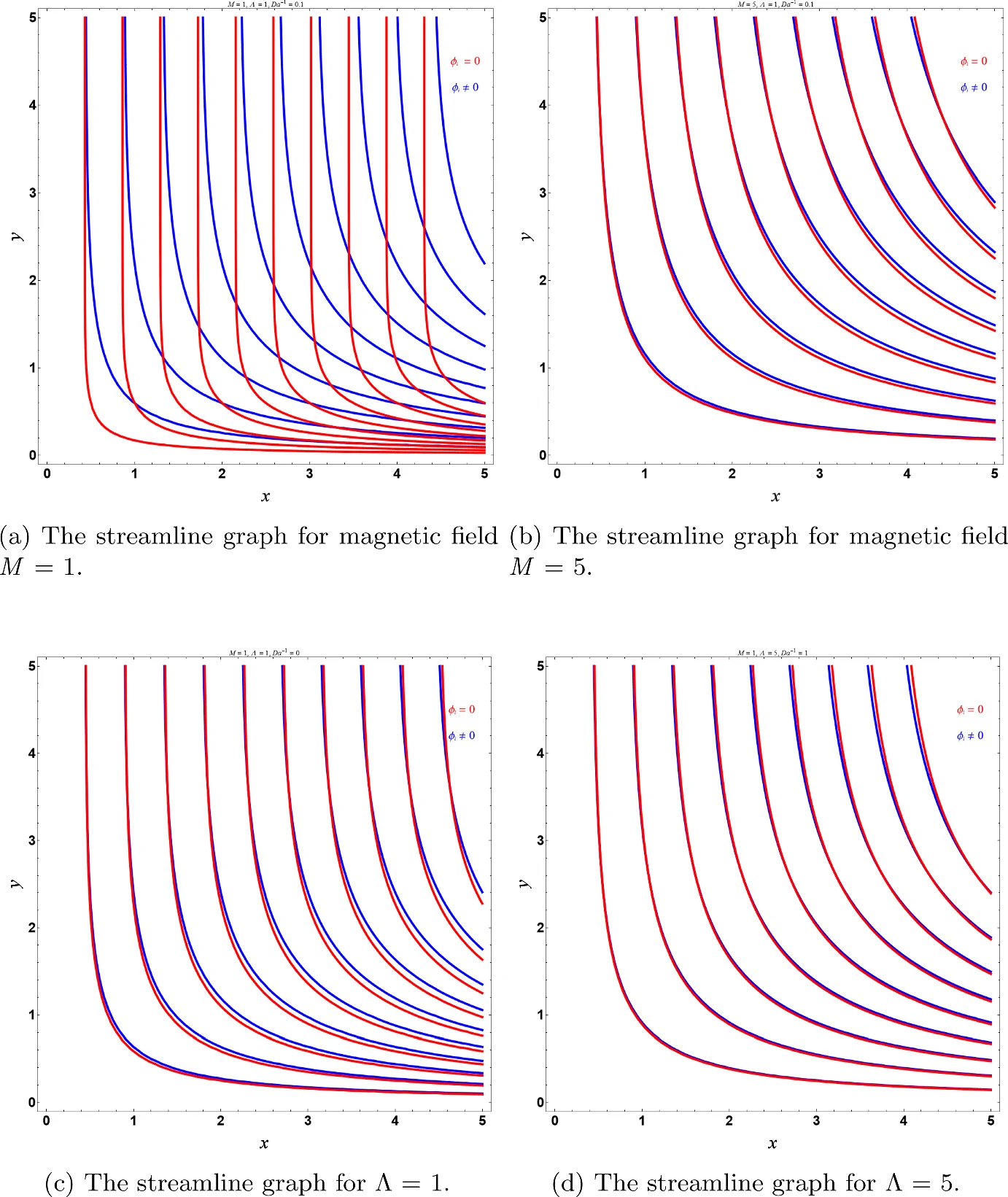
Comparison of streamline behaviour in the (*x*, *y*) plane for $\phi _i = 0$ (red curves) and $\phi _i \ne 0$ (blue curves) under varying parameter regimes : (**a**) and (**b**) Magnetic Parameter M and (**c**) and (**d**) effective porous medium viscosity

Figure 11 presents a comparative analysis of the system’s behaviour under two conditions: $\phi _i = 0$ (red curves) and $\phi _i \ne 0$ (blue curves), across four parameter regimes. Each subplot visualises the spatial evolution of the field in the (*x*, *y*) plane, highlighting how the presence of nanoparticles alters the streamline structure. In all cases, the blue curves exhibit greater curvature compared to the red curves, indicating that non-zero $\phi _i$ introduces stronger gradients and more pronounced boundary-layer effects. As *M* increases (e.g., M=5 in subfigure b), the magnetic field intensifies Lorentz damping, which interacts with the nanoparticle-induced changes to further distort the field lines. Similarly, variations in the viscosity ratio Λ modify the velocity decay rate, while variations in $Da^{-1}$ alter the porous-medium resistance; both effects influence the curvature and spread of the streamlines. The consistent divergence between $\phi _i = 0$ and $\phi _i \ne 0$ across all subplots confirms that nanoparticle volume fraction plays a significant role in shaping the system’s spatial dynamics, especially in magnetised porous environments.

Overall, the observed parametric trends can be physically interpreted as follows. Increasing the magnetic parameter *M* strengthens the Lorentz force, which suppresses the fluid velocity and modifies the convective transport of thermal energy. Accordingly, the resulting changes in the temperature field arise indirectly through the coupling between the momentum and energy equations rather than through an explicit Joule-heating term. Similarly, increasing the inverse Darcy number $Da^{-1}$ enhances the resistance imposed by the porous medium, reducing the fluid velocity and thereby altering convective heat transport. No explicit Darcy-dissipation term is included in the present energy equation. The nanoparticle volume fraction modifies the effective thermophysical properties of the fluid, while the thermal radiation parameter *Nr* directly influences the energy equation through the radiative heat-flux contribution and therefore affects the thermal boundary-layer thickness and temperature distribution.

### A Model Benchmark Example

For independent numerical validation of the analytical solutions, the reduced boundary-value problems were solved using MATLAB’s bvp4c solver on a truncated similarity domain.

For validation of the analytical velocity solution, we consider the limiting case $$A_1=A_2=A_3=\Lambda =1$$ and $$Da^{-1}=0$$. The quantity compared is the normalized wall velocity gradient $-f^{\prime \prime }\left(0\right)$. From Eqs. (28)–(29),

60 $$\begin{aligned} -f''(0)=\delta =\sqrt{\frac{A_2+A_3M+A_1Da^{-1}}{\Lambda }}, \end{aligned}$$

which, under the above limiting conditions, reduces to

61 $$\begin{aligned} -f''(0)=\sqrt{1+M}. \end{aligned}$$

An independent numerical solution of the same reduced momentum boundary-value problem was obtained and compared with the analytical result for different values of the magnetic parameter *M*.

**Table 4:** Comparison of the normalized wall velocity gradient $-f^{\prime \prime }\left(0\right)$ obtained from the present analytical solution and an independent numerical BVP solution for $$A_1=A_2=A_3=\Lambda =1$$ and $$Da^{-1}=0$$

*M*	Numerical BVP solution	Present analytical solution	Absolute difference
0.0	1.00000113	1.00000000	$1.13\times 10^{-6}$
0.2	1.09544497	1.09544512	$1.50\times 10^{-7}$
0.5	1.22474467	1.22474487	$2.00\times 10^{-7}$
1.0	1.41421299	1.41421356	$5.70\times 10^{-7}$
1.2	1.48323902	1.48323970	$6.80\times 10^{-7}$
1.5	1.58113805	1.58113883	$7.80\times 10^{-7}$
2.0	1.73204996	1.73205081	$8.50\times 10^{-7}$

Table 4 shows excellent agreement between the independent numerical BVP solution and the present analytical solution for the normalized wall velocity gradient $-f^{\prime \prime }\left(0\right)$. Across the considered range $$0\le M\le 2$$, the maximum absolute difference is $$1.13\times 10^{-6}$$, occurring at M=0, while all remaining differences are below $$10^{-6}$$. The numerical values therefore reproduce the analytical dependence

62 $$\begin{aligned} -f''(0)=\sqrt{1+M}, \end{aligned}$$

to high accuracy. This independent numerical comparison validates the closed-form velocity solution for the limiting case considered and confirms the predicted dependence of the normalized wall velocity gradient on the magnetic parameter.

To independently validate the closed-form temperature solution, we consider the limiting case $$A_1=A_2=A_3=A_4=A_5=\Lambda =1$$, $$Nr=M=Da^{-1}=0$$, and Pr=6.21. The normalized wall-temperature gradient $-\theta^{\prime }\left(0\right)$ obtained from the numerical temperature boundary-value problem is compared with the corresponding analytical result for different values of the thermal relaxation parameter γ.

**Table 5:** Comparison of the normalized wall-temperature gradient obtained from the present analytical solution and an independent numerical BVP solution for $A_1=A_2=A_3=A_4=A_5=\Lambda =1$, $Nr=M=Da^{-1}=0$, and Pr=6.21

γ	Numerical BVP solution	Present analytical solution	Absolute difference
0.00	1.77255147	1.77255111	$3.60\times 10^{-7}$
0.02	1.77989932	1.77989918	$1.40\times 10^{-7}$
0.05	1.79102056	1.79102066	$1.00\times 10^{-7}$
0.10	1.80981512	1.80981527	$1.50\times 10^{-7}$
0.15	1.82892180	1.82892199	$1.90\times 10^{-7}$

Table 5 demonstrates excellent agreement between the present analytical solution and the independent numerical BVP solution for all considered values of the thermal relaxation parameter γ. The maximum absolute difference between the two solutions is $3.60\times 10^{-7}$, occurring at γ=0, while all remaining differences are below $2\times 10^{-7}$. This close agreement provides an independent numerical validation of the derived closed-form temperature solution for the limiting case considered and confirms consistency with the reduced energy equation and its associated boundary conditions.

## Conclusions

This work has examined the combined influence of thermal radiation, viscosity ratio, and magnetohydrodynamic forces on the flow and heat transfer of a water based tetra hybrid nanofluid over a stretching surface embedded in a porous medium. By incorporating the viscosity ratio and MHD effects into the momentum equation and radiation into the energy equation, and by comparing di-, tri-, and tetra-hybrid nanoparticle formulations, the study addresses a clear gap in the existing literature. Through similarity transformations, the governing equations were reduced to ordinary differential equations, which were solved analytically using Appell hypergeometric functions. Parametric behaviour was subsequently explored through detailed graphical analysis. Unlike most existing studies on hybrid and tetra-hybrid nanofluids, which rely primarily on numerical or semi-analytical techniques, the present work provides closed-form analytical solutions that simultaneously incorporate viscosity ratio, magnetohydrodynamic forces, thermal radiation, and non-Fourier heat flux effects, extending earlier MHD formulations reported in [15, 17, 32, 33].

The key findings can be summarised as follows. Increasing the magnetic field strength suppresses both axial and transverse velocities due to enhanced Lorentz damping. In contrast, increasing the viscosity ratio decreases the velocity-decay parameter, resulting in larger axial and transverse velocities and a thicker momentum boundary layer. Increasing the inverse Darcy number suppresses velocity through enhanced porous-medium resistance. Thermal behaviour shows the opposite trend: stronger radiation, higher viscosity ratio, and larger magnetic parameters all thicken the thermal boundary layer and elevate the temperature field. Likewise, increasing the nanoparticle volume fraction modifies the effective thermal properties and leads to changes in the temperature field and thermal boundary-layer characteristics. The observed suppression of axial and transverse velocities with increasing magnetic parameter and inverse Darcy number is consistent with classical Darcy and MHD boundary-layer theories, where Lorentz forces and porous-medium drag act as dominant resistive mechanisms [15, 18, 32].

These results highlight the potential of tetra-hybrid nanofluids for advanced thermal management applications, including electronics cooling, high performance industrial heat exchangers, thrust bearing systems, polymer processing, and biomedical thermal regulation. The comparative analysis across di-, tri-, and tetra- hybrid formulations provides valuable insight into how increasing particle complexity influences both momentum and heat transfer. The inclusion of thermal radiation also underscores the relevance of this model to high temperature porous media environments such as combustion chambers and energy storage systems. The thickening of the thermal boundary layer with increasing radiation parameters aligns with earlier studies on radiative nanofluid transport in porous and magnetised environments [21–25, 37]. These results are particularly relevant to high-temperature and controlled thermal systems, including porous heat exchangers, polymer processing, and biomedical hyperthermia applications, where radiative effects and magnetic nanoparticle transport play a critical role [36, 49, 50].

The study does, however, have limitations. The mathematical model is idealised and may not capture all complexities of real nanofluid suspensions, and the specific nanoparticle combinations considered may not generalise to all base fluids or operating conditions. Practical implementation may be constrained by nanoparticle cost, stability, and environmental considerations, and scaling laboratory-scale predictions to industrial systems remains non trivial. The contribution of this work lies primarily in the analytical formulation and comparative study of multi-component nanofluids, rather than in identifying new physical trends.

Future work could extend the present framework to incorporate additional physical effects and more complex flow configurations. Possible directions include viscoelastic [51] or non-Newtonian fluid rheology, buoyancy-driven convection, convergent–divergent geometries, and exponential or nonlinear stretching surfaces. The model could also be adapted to flows over Riga plates, in which appropriately arranged electrodes and permanent magnets generate a Lorentz force that can be used to control the near-wall flow, as well as to confined microchannel configurations relevant to microscale transport applications. Such extensions would broaden the applicability of the model and enable exploration of a wider class of technologically relevant flow systems.

## Data Availability

Data sharing is not applicable to this article as no datasets were generated or analysed during the current study.
